# The Emerging Role of Branched-Chain Amino Acids in Insulin Resistance and Metabolism

**DOI:** 10.3390/nu8070405

**Published:** 2016-07-01

**Authors:** Mee-Sup Yoon

**Affiliations:** Department of Molecular Medicine, School of Medicine, Gachon University, Incheon 406-840, Korea; msyoon@gachon.ac.kr; Tel.: +82-32-899-6067; Fax: +82-32-899-6039

**Keywords:** branched-chain amino acids (BCAAs), insulin resistance, mammalian target of rapamycin complex 1 (mTORC1), metabolism

## Abstract

Insulin is required for maintenance of glucose homeostasis. Despite the importance of insulin sensitivity to metabolic health, the mechanisms that induce insulin resistance remain unclear. Branched-chain amino acids (BCAAs) belong to the essential amino acids, which are both direct and indirect nutrient signals. Even though BCAAs have been reported to improve metabolic health, an increased BCAA plasma level is associated with a high risk of metabolic disorder and future insulin resistance, or type 2 diabetes mellitus (T2DM). The activation of mammalian target of rapamycin complex 1 (mTORC1) by BCAAs has been suggested to cause insulin resistance. In addition, defective BCAA oxidative metabolism might occur in obesity, leading to a further accumulation of BCAAs and toxic intermediates. This review provides the current understanding of the mechanism of BCAA-induced mTORC1 activation, as well as the effect of mTOR activation on metabolic health in terms of insulin sensitivity. Furthermore, the effects of impaired BCAA metabolism will be discussed in detail.

## 1. Introduction

Branched-chain amino acids (BCAAs) are critical nutrient signals that affect metabolism, either directly or indirectly. The BCAAs comprise leucine, isoleucine, and valine, which are essential amino acids [1]. BCAAs are comparatively abundant in dietary proteins, constituting up to 15%–20% of protein intake, which increases after intake of a meal containing protein [2]. It has been shown that a positive association exists between a BCAA-rich diet and metabolic health, including the regulation of body weight, muscle protein synthesis, and glucose homeostasis [2,3]. In spite of the positive effects of BCAAs on metabolic health, an elevation in the level of BCAAs correlates with an increasing risk of insulin resistance (IR) and type 2 diabetes mellitus (T2DM) in humans and in rodent models [4]. This paradoxical role of BCAAs in metabolism raises some questions. First, are BCAAs and a BCAA-rich diet beneficial or harmful in terms of insulin and glucose homeostasis? Second, are BCAAs causative or predictive of insulin resistance? Third, what induces an elevation in the level of BCAAs in the insulin resistant state? After addressing these questions, this review describes the recently accumulated studies that show the potential role of BCAAs in insulin signaling. In addition, a possible mechanism underlying the elevation BCAAs in insulin resistant states and the role of BCAAs in insulin signaling will also be discussed in the following section.

## 2. The Positive Effects of BCAAs on Metabolic Health

Even though the association between a high level of BCAAs and IR has been shown in numerous studies in humans and in rodent models, elevating the level of BCAAs leads to positive effects that improve metabolic parameters such as body composition, glycaemia levels, and satiety.

Hypothalamic leucine is a potential nutrient signal that may reduce food intake by activating mammalian target of rapamycin (mTOR) [5]. mTOR is activated robustly and selectively within mediobasal hypothalamic (MBH) arcuate nucleus (ARC) neurons expressing anorexigenic, pro-opiomelanocortin (POMC), and orexigenic neuropeptide Y/agouti-related peptide neurons within the ARC during refeeding after a fast [5]. The leucine level in the MBH engages forebrain/hindbrain neurocircuitry that provides negative feedback to energy balance by lessening food intake [6]. Cota et al. suggested that these hypothalamic BCAA sensitive responses maintain organisms in a state of metabolic balance [5].

BCAAs control hormone release in both the gastrointestinal tract and in fat deposits. Treatment with leucine for six weeks increased adiponectin and decreased cholesterol in the plasma of previously obese mice, without changing body weight or fat mass [7]. BCAAs and dietary protein enhanced glucagon like peptide-1 (GLP-1) release and lowered the expression levels of the genes required for synthesis and adsorption of fatty acids in a human intestinal cell line (NCI-H716), suggesting an intestinal mechanism for the beneficial effect of BCAAs [8]. In addition, the elevated BCAA levels induced insulinemic responses and insulintropic effects in mice [9,10].

Together with insulin, BCAAs also function as anabolic signals to alter the growth of energy-consuming tissues such as skeletal muscle and adipose tissue. Amino acids are required for protein synthesis. Amino acids, BCAAs in particular, also stimulate mTORC1 signaling pathways to regulate mRNA translation [11]. Leucine-activated mTOR augments the eIF4E-eIF4G complex by increasing the availability of eIF4E and by phosphorylating eIF4G, resulting in accelerated protein synthesis [11]. Oral administration of leucine in Sprague–Dawley rats augments protein synthesis in adipose tissue, gastrocnemius muscle, and kidney, but not in the liver or the heart. Conversely, carbohydrate meals did not alter protein synthesis in any tissue. Rather, it prompted a robust insulin increase, suggesting that leucine is a direct nutrient signal, which induces protein synthesis [12]. Moreover, leucine partially inhibits muscle atrophy by reducing proteolysis and autophagy [13]. Amino acid-induced protein synthesis shows a positive correlation with energy expenditure [14]. BCAA supplements can have a beneficial effect on certain liver diseases by stimulating protein synthesis, and the secretion of hepatic growth factor, and by inhibiting proteolysis [15]. Dietary leucine supplement in the drinking water (1.5% *w*/*v*) doubled leucine level in the plasma of high fat diet fed mice, leading to improvement of glucose tolerance and insulin sensitivity, as well as decrease in hepatic steatosis and in inflammation of adipose tissue without affecting food intake and weight gain [16].

Taken together, both BCAA-rich diets and BCAA supplementation have positive roles in metabolism, supporting the dietary recommendation for protein to increase BCAA levels.

## 3. The Negative Effects of BCAAs on Metabolism

Recent metabolomics studies and comprehensive metabolic profiling studies have consistently showed a disturbance of normal amino acid metabolism and an increase of specific amino acids, often BCAAs, in some rodent models of obesity or T2DM, and in patients with obesity or T2DM [17,18,19,20,21]. Considering the above cited health benefits of BCAAs, the correlation of high levels of BCAAs with insulin resistance, obesity and T2DM seems to be contradictory.

Newgard et al. showed that BCAAs, aromatic amino acids, and BCAA byproducts are most strongly correlated with insulin sensitivity and the homeostasis model assessment-insulin resistance index (HOMA-IR) than many other lipid species [5]. They measured more than 100 analytes from both obese, insulin-resistant patients and lean, insulin-sensitive subjects, followed by principal component analysis (PCA). In addition, the preferential association of BCAAs with insulin resistance was demonstrated in normal weight healthy subjects [22]. Measurement of 191 metabolites by mass spectrometry revealed that the decrease in the level of BCAAs, together with that of glycerol, is strongly predictive of insulin sensitivity. In line with these results, Hattersley et al. showed that fat-reduced diet modulated AA and BCAA metabolic signature under an isoenergetic diet in the absence of weight change, implying that AA metabolic signatures can be used to assess the diabetic risk [23]. The association between BCAA signature and IR was supported by Fiehn’s study which showed that leucine and valine among >350 metabolites were increased in African-American T2DM women subjects [24]. It was further confirmed in a cross-sectional study of 73 overweight/obese individuals without diabetes by performing glucose tolerance tests to measure insulin sensitivity [19]. Moreover, several longitudinal studies in different cohorts have revealed that increased blood levels of BCAAs anticipate future likelihood of developing insulin resistance or T2DM [3,25].

It has been suggested that BCAAs influence brain function by competing with the uptake of the amino acid precursors of dopamine and 5-hydroxytryptamine in the brain [26]. Large neutral amino acid (LNAA) transport is shared by BCAAs and aromatic amino acids, leading them to compete with each other. The elevation in the level of BCAAs reduces the aromatic amino acid level, leading to a reduction in the synthesis and release of neurotransmitters derived from aromatic amino acids, possibly contributing to an increased risk of depression [2].

Despite the positive effects of BCAAs on metabolism, the strong association of BCAA levels with insulin resistance and metabolic syndrome suggests that increased levels of BCAAs may cause insulin resistance and T2DM, although this remains a speculation for now. The mechanism underlying that correlation is not yet fully understood. The possible mechanism by which BCAAs contribute to IR will be discussed in the following section.

## 4. Mechanism of IR by mTOR

Even though it is not still clear whether BCAAs are causative factors in the development of IR, or whether they are biomarkers of impaired insulin action, the involvement of nutrient signaling in insulin resistance has emerged. High levels of BCAAs persistently activate mTORC1 (mTOR complex 1), resulting in insulin resistance through the phosphorylation of insulin receptor substrate 1 (IRS-1).

### 4.1. The Mechanism of Amino Acid-Induced mTORC1 Activation

mTOR is a serine/threonine kinase belonging to the phosphatidylinositol (PI) kinase-related protein kinase family [27]. The mTOR signaling network regulates critical cellular and developmental processes such as cell growth, differentiation, cell survival, and metabolism [28]. mTOR exists in at least two biochemically and functionally distinct complexes, mTOR complex 1 (mTORC1) and mTOR complex 2 (mTORC2) [29]. mTORC1 regulates cell growth in response to several extracellular and intracellular signals, including growth factors, cellular energy status, cellular oxygen level, and amino acid availability. mTORC1 consists of “regulatory-associated protein of mTOR” (raptor), which recruits substrates and helps mTOR localization; “40 kDa Pro-rich Akt substrate” protein (PRAS40), “DEP domain-containing mTOR-interacting” protein (DEPTOR), all of which are negative regulators of mTORC1; and “mammalian lethal with SEC13 protein 8” (mLST8; also known as GβL), which positively regulates mTORC1. Two most well-known down stream targets of mTORC1 are 4EBP1 and S6K1, which regulate mRNA translation initiation and progression, consequently protein synthesis [28].

Tuberous sclerosis complex 1/2 (TSC1/2) transduces the upstream signal to mTORC1 [30]. TSC is a guanosine triphosphate (GTP) ase-activating protein (GAP) for the small GTPase Rheb (Ras homologous enriched in brain), which negatively regulates mTORC1 by increasing the GTP hydrolysis rate of Rheb. However, amino acid signaling activates mTORC1 independently of TSC regulation, by regulating intracellular mTORC1 localization [31]. During amino acid deficiency, mTORC1 diffuses throughout the cytoplasm. Upon amino acid stimulation, mTORC1 translocates to the lysosomal surface, where the small GTPase Rheb resides [29]. Zoncu et al. reported that amino acids accumulate in the lysosome after their extracellular addition, suggesting that the lysosome is a key site of amino acid-induced mTOR activation [32].

Compared to other mTOR upstream signals, amino acids do not impinge on the TSC-Rheb axis. The Rag GTPase-Regulator axis mediates amino acid signal to mTORC1 [33]. Four members of the Rag GTPases family function as heterodimers (Rag A/B and Rag C/D) in mammalian systems. Rag proteins on the lysosome recruit raptor, depending on the nucleotide-bound state of the Rags (Rag A/B GTP bound, and Rag C/D GDP bound), which are regulated by amino acid availability [31]. Ragulator, a pentameric complex, functions as a lysosomal tether for Rag proteins through myristoylation and palmitoylation of “Late endosomal/lysosomal adaptor, mitogen activating protein kinases (MAPK) and mTOR activator 1” (Lamtor 1) [34]. Moreover, Ragulator also functions as a guanine exchange factor (GEF) for both Rag A and Rag B by preferentially binding to them in the nucleotide-free state [34]. In the absence of amino acids, Ragulator interacts with V-ATPase, a vacuolar proton pump on lysosomes. This interaction blocks the GEF activity of Ragulator toward Rag proteins [32]. Amino acids induce conformational changes in V-ATPase, releasing and activating Ragulator [32]. Additionally, folliculin (FLCN), a tumor suppressor, together with its binding partner FNIP1 (folliculin interacting protein1) has been suggested to be a GAP for Rag C and Rag D [35]. Leucyl-tRNA synthetase (LRS) has also been shown to bind to Rag D, and function as a GAP for Rag D, in a leucine dependent manner [36].

A recent report identified GATOR complexes as negative regulators of Rag proteins. GATOR1 interacts directly with Rag proteins, functioning as a GAP toward Rag A and Rag B, leading to inhibition of mTORC1 activation [37]. GATOR2 inhibits GATOR1, blocking inhibitory regulation of mTORC1, resulting in mTORC1 activation [37]. Notably, Sestrin 2, a leucine sensor, also binds to GATOR2, thereby inhibiting mTORC1 in the absence of leucine, even though how its interaction inhibits mTORC1 is unknown [38]. Finally, it has been shown that leucine binds directly to Sestrin 2, disrupting the interaction with GATOR2, and activating mTORC1 [39].

The identification of amino acid mediators on mTORC1 translocation to the lysosome surface notwithstanding, the Vps34-phospholipase D1 (PLD1) axis was also suggested to activate mTORC1 on the lysosome [40]. Phosphatidic acid (PA), a product of phospholipase D1, is known to be a critical regulator of mTOR activation. PA binds to the FKBP rapamycin binding (FRB) domain of mTOR, competing with rapamycin and activating mTOR [41]. In addition, PLD1-produced PA is responsible for mTOR activation during both amino acid- and mitogen-induced mTOR activation [42]. Recently, PI3P, a Vps34 product, has been found to activate PLD1, promoting its translocation to the lysosome, independently of the Rag-Ragulator complex [40]. PLD1 produces PA on the lysosome surface, activating mTOR, in addition to Rag-induced mTOR regulation. Although the involvement of Ca^2^^+^-calmodulin during Vps34 activation has been suggested, the upstream regulators that activate Vps34 remain unknown.

### 4.2. The Proposed Mechanism Underlying IR by BCAAs-Induced mTORC1 Activation: Some of the Possible Mechanisms

**Figure 1:**
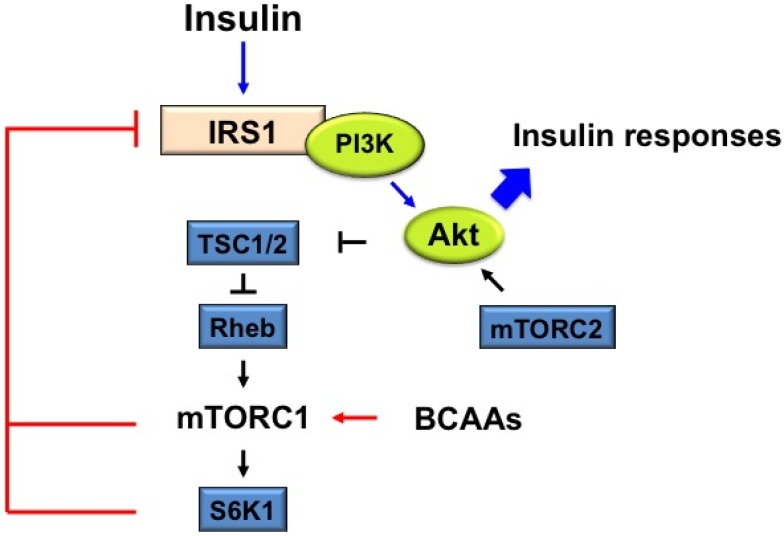
The proposed mechanism of branched-chain amino acids (BCAAs)-stimulated mammalian target of rapamycin complex1 (mTORC1) activation on insulin resistance (IR). BCAAs-activated mTORC1 and following S6K1 phosphorylate insulin receptor substrate 1 (IRS-1) at serine 307, 636/639, 1101, 312, which inhibit IRS-1. Impaired protein kinase B (PKB, also known as Akt) activation via the negative feedback regulation attenuates insulin responses, such as the increase of glucose uptake and glycogen synthesis and decrease of glucose synthesis.

mTOR is a central signaling mediator of crosstalk between amino acids and insulin. Insulin activates mTOR, followed by activation of ribosomal S6K1, which regulates initiation of translation and elongation by phosphorylating S6 [43]. Either activated mTOR or activated S6K1 phosphorylate IRS-1, leading to blocked insulin signaling [44,45] (Figure 1).

Insulin triggers autophosphorylation of the insulin receptor, leading to the recruitment and phosphorylation of IRS1/2 at multiple tyrosine residues [46]. These sites function as docking motifs for the P85 regulatory subunit of class I phosphoinositide 3-kinase (PI3K), followed by phosphatidylinositide-3,4,5-P3 (PIP3), which binds to the pleckstrin homology (PH) domain of Akt, which leads in turn to the phosphorylation of Akt on threonine 308 in activation loop by phosphatidylinositide dependent kinase 1 (PDK1) [27,47]. Akt also is phosphorylated on serine 473 in C-terminal hydrophobic motif by mTORC2 [27]. Akt activates mTOR/S6K1 by phosphorylating TSC1/2. Notably, hyperactive S6K1 and mTOR negatively regulate Akt by inducing IRS-1 serine phosphorylation (S302 (human 307) [48], S636/639 [49], S1101 [50], S307 (human 312) [17,51]), which disrupts its interaction with the insulin receptor, and results in degradation. In insulin resistant subjects, such as those with obesity and T2DM, a chronically high level of amino acids could maintain the hyperactivation of mTOR/S6K1, which works together to establish a negative feedback loop. Indeed, it has been shown that approximately 2.1 fold increase of plasma amino acids by amino acid infusion increased insulin-stimulated mTORC1/S6K1 activity in humans, followed by the phosphorylation of IRS-1 S307 (human 312) and S636/639 [52].

## 5. The Controversy over the Role of mTORC1 in IR

Despite several lines of experimental evidence supporting the view that the effect of BCAAs on insulin resistance occurs through mTORC1 activation, some observations are controversial.

Increasing the level of BCAAs, by supplementation or genetic modification, correlates with an improvement in metabolism, in spite of activated mTORC1 signaling. Deletion of mitochondrial branched-chain aminotransferase (BCATm) increased energy expenditure and improved insulin sensitivity [53]. BCATm catalyzes the transfer of an α-amino group from a BCAA to α-ketoglutarate, forming glutamate and the three respective branched-chain α-keto acids. Depletion of BCATm elevates BCAA levels 10 times relative to wild type mice due to the blockage of BCAA catabolism. Unexpectedly, these mice consumed more food and exhibited increased diet-induced thermogenesis (DIT), followed by increased rates of protein synthesis and degradation, accompanied by a lean phenotype. These results suggest that elevated levels of BCAAs in mice with deficient BCAA catabolism arise from an increased protein turnover rate, leading to an increased energy expenditure.

In addition, it is not clear whether the small changes in the BCAA level could increase IRS-1/2 serine phosphorylation under physiological conditions. Since many studies administrated larger doses of leucine compared to the BCAA levels in diabetic patients, it has not been shown that physiological levels of BCAAs can induce mTOR activation and the subsequent serine phosphorylation of IRS1 and IRS2. In line with this, Weickert et al. observed the only transient high-protein diets affect insulin sensitivity [54]. Under isoenergetic diet and weight-maintaining diet, a high protein diet for six weeks increased S6K1, an mTORC1 downstream target, confirming association AA levels with IR. However, after a long intervention (6 weeks–18 weeks), high protein diets no longer affect S6K1 expression, resulting in diminishing the effect on insulin sensitivity. This implied that mTORC1 might play a role in IR under the changes of BCAA levels for the short-term. Magkos et al. found that mTOR activity was not altered following gastric bypass surgery in a morbidly obese individual, despite alleviation of BCAA levels, supporting the dissociation between BCAA levels and mTOR activation [55]. Even in Newgard’s work, high dietary BCAAs level did not alter mTOR activation and insulin sensitivity in regular chow diet feeding, suggesting that the association of BCAAs with IR was evident in high fat diets [17,56]. Indeed, BCAA-fed mice in the drinking water manifested high mTOR activity without affecting insulin sensitivity, suggesting the dissociation between BCAAs-elicited mTOR activity and IR [57]. Taken together, the association between mTORC1 activation and IR is needed further investigate to get a clear insight into their relation.

## 6. The Processes That Affect the Level of BCAAs

In order to understand the potential role of BCAAs on metabolism, it would be beneficial to understand the processes that promote high blood levels of BCAAs. Considering that BCAAs are essential amino acids that cannot be synthesized de novo in organisms, the level of circulating BCAAs could be contributed to by dietary intake and by degradation of protein in tissue. This idea is supported by the fact that BCAAs regulate protein degradation by diminishing autophagy and proteasomal activity. BCAAs impair autophagy by activating mTORC1, a negative regulator of the initiation of autophagy. In agreement with this view, leucine restriction promotes protein breakdown in muscle cells by inhibiting mTOR, leading to an induction of autophagy [58]. Dietary leucine dampens protein degradation in the muscles of rats fed a protein-deficient diet by regulating autophagy, without affecting protease activity or ubiquitin ligase mRNA expression [59], confirming a critical role for leucine-regulated autophagy in the degradation of muscle. Leucine also elicits the ubiquitination of certain proteins, leading to their degradation. Leucine supplementation reduced mass loss from the soleus muscle during hind limb immobilization by attenuating the expression of E3 ligase, muscle ring fiber 1 (MuRF1), and muscle atrophy F-box (MAFbx)/atrogin-1, followed by a decrease in ubiquitinated proteins [60]. Regardless of the pivotal role played by BCAAs in protein degradation via both autophagy and proteosomal degradation, increased dietary protein intake might not be a main reason for the abnormally high levels of BCAAs in obese and insulin resistant subjects. The level of BCAAs is elevated in obesity, even following an overnight fast [61,62], and dietary uptake in insulin resistant subjects is comparable to that in insulin-sensitive subjects in either Asian-Indian or Chinese patients [63], suggesting that the abnormally high BCAA levels could be provoked by mechanisms other than degradation of dietary protein.

Protein turnover is controlled by insulin, in addition to amino acids. Although insulin activates protein synthesis in newborn piglets [64], the enhanced protein synthesis in humans under conditions of hyperaminoacidemia is attributable to insulin reducing the rate of protein degradation [65]. In accordance with this, insulin reduces the mRNA expression of MAFbx and proteasome C2 subunit proteins in human muscle [66]. The availability of insulin and amino acids activates protein synthesis additively, suggesting that amino acids and insulin regulate protein synthesis independently [66]. This could account for the observation that protein degradation becomes increased in fasting individuals with obesity and insulin resistance, without affecting protein synthesis [67,68,69]. Although BCAAs do not affect protein degradation directly, BCAAs might play a critical role in modulating muscle mass when insulin-induced protein degradation becomes decreased by insulin resistance [2].

In addition, gut microbiota influence BCAAs level in the plasma [70]. Gut microbiota put to use several amino acids including BCAAs from host to either synthesize bacterial cellular components, or catabolizing them to generate metabolic products such as short chain fatty acid (SCFA) and branched-chain fatty acids (BCFA) which plays a role in development of obesity. Notably, gut bacteria raises amino acids level by either de novo biosynthesis [71], or affecting nutrient absorption [72]. Even though gut microbes have been shown to contribute to amino acid levels in hosts, the importance of gut microbes in BCAA levels need to be assessed further.

## 7. BCAA Dysmetabolism

Along with persistent mTORC1 activation, impairments in BCAA metabolism are linked to insulin resistance and T2DM by the accumulation of possibly toxic intermediates and BCAA levels in plasma.

The mitochondrial isoform of branched-chain aminotransferase (BCATm, encoded by *BCAT2*) catalyzes the first step in the metabolism of BCAAs in most peripheral tissues. BCAA metabolites are diminished in the peripheral tissues of *BCAT2-/-*mice [53], as mentioned in the previous section. Despite their continual activation of mTORC1, *BCAT2-/-*mice do not have insulin resistance, which would be expected. Instead, *BCAT2-/-*mice exhibit ameliorated glycemic control and insulin sensitivity with high energy expenditure, probably due to the loss of gluconeogenic precursors, indicating that muscle transaminases play a critical role in the generation of gluconeogenic substrates for the liver.

The next step in the BCAA metabolic pathway is catalyzed by the multienzyme mitochondrial branched-chain α-ketoacid dehydrogenase complex (BCKDC) [2]. BCKDC oxidizes BCAAs irreversibly to their respective ketoacids. Importantly, the expression and activity of BCKDC can be altered by numerous metabolic factors, which are related to obesity, insulin resistance, and T2DM. Mutation of BCKDC and its activator, the mitochondrial isoform of protein phosphatase 1K (PPM1K), result in accumulation of BCAAs and branched-chain α-ketoacids (BCKAs), followed by maple syrup urine disease (MSUD) [73,74,75]. In fact, treatment of glial cells, the cerebral cortex, or rat liver cells, with several BCKAs, or with the α-ketoacid of leucine, α-ketoisocaproate (α-KIC), resulted in mitochondrial dysfunction [73,76,77]. Branched-chain acyl-Coenzyme A (CoA) species are produced by BCKDC. These are further metabolized by multiple enzymatic steps within the mitochondrial-matrix, eventually forming lipogenic, ketogenic, or glucogenic substrates (acetoacetyl-CoA, acetyl-CoA, and propionyl-CoA).

In the adipose tissue of patients with obesity and T2DM with insulin resistance, the expression of genes encoding the enzymes of BCAA metabolism is significantly decreased through an undefined mechanism—at least those genes encoding enzymes catalyzing the first two steps—leading to an increased plasma level of BCAAs [78,79]. Considering that whole-body BCAA metabolism is substantially interorgan-dependent, the expression of these enzymes in other organs, such as the liver and the muscles, needs to be considered. The expression of the genes encoding the enzymes of BCAA metabolism was reduced in muscle and liver tissue of patients with T2DM [80,81]. Similar findings were made in rats [82]. In contrast, liver BCKDH activity is actually increased and could compensate for the decreased activity in adipose tissue [83]. Therefore, the resulting plasma BCAA levels are either elevated or unchanged, depending on the enzymatic activity in other organs.

Altered gene expression, caused by either mutations or epigenetic regulation, affects all the enzymatic activities of BCAA metabolism. Tiffin et al. identified BCKDH4, the gene encoding the regulatory subunit of BCKDC, as one of two primary susceptibility genes that affect the risk of developing both T2DM and obesity via computational disease prioritization methods [84]. Furthermore, that same study found IVD to be a secondary T2DM susceptibility gene. IVD encodes isovaleryl-CoA dehydrogenase, which is involved in leucine metabolism [84]. PPM1K, the BCKDHA phosphatase, was chosen as one of the top 20 candidate genes for a T2DM study [85]. Altered BCKDC activity achieved by acute exercise [86] or the regulator BCKA [87], could also modulate plasma BCAA levels, either in the short-term or the long-term. Among several factors, long-chain fatty acids and their metabolites impeded BCKDC activity either directly, by affecting either redox states or the concentrations of acetyl-CoA, or indirectly by inhibitory carbonylation of enzymes, possibly causing BCAA dysmetabolism [88,89,90,91].

## 8. Conclusions

The association between the level of circulating BCAAs, insulin resistant obesity, and T2DM prompted consideration of BCAA levels as a predictor for future insulin resistance or T2DM, in spite of the beneficial effects of BCAA supplementation and a BCAA-rich diet. Elevated BCAAs stimulate mTORC1, a nutrient sensing complex, and following IRS-1 serine phosphorylation, result in insulin resistance and other metabolic disorders. However, recent works sparked questions on whether mTOR activation by physiological changes in the level of BCAAs is sufficient or necessary to cause insulin resistance, and subsequent metabolic disorders. The correlation between BCAAs level and insulin resistance was matched to HOMA-IR before diet intervention, but not after [92]. Recent work also exhibited using untargeted metabolomics that BCAA levels were not elevated in a UC Davis (UCD)-T2DM rat model until six months post-onset of diabetes [93]. These suggested that the increase of BCAAs level is not enough to elicit IR and T2DM in rat model systems. Impairment of BCAA metabolism also contributes to increased levels of BCAAs in insulin resistant subjects. It has been suggested that accumulation of BCAAs promotes mitochondrial dysfunction, linked to stress kinase stimulation and β-cell apoptosis [2], which are frequently related to insulin resistance and T2DM. On the other hand, incomplete oxidation of BCAAs might cause an imbalance between anaplerosis and cataplerosis, triggering suboptimal mitochondrial functioning. For example, reduction of mitochondrial BCKD activity elicits accumulation of BCKA and α-ketobutyrate (α-KB) resulting in restriction of propionyl-coA-derived metabolites into tricarboxylic acid (TCA) cycles, inducing anaplerotic stress and diminished amino acid fuel delivery to mitochondria [24,56,94]. Hence, the role played in metabolism by high levels of BCAAs warrants further study.

In summary, recent studies propose a close association between BCAAs and insulin resistance. Mechanisms whereby increased BCAAs induce insulin resistance have been proposed. The new findings relating to the BCAA signaling pathway, and to BCAA metabolism, broaden our understanding of insulin resistance. However, whether BCAAs are simply markers of insulin resistance, or whether they are direct contributors to insulin resistance remains uncertain, and this issue is attracting increased research interest. Furthermore, the signaling pathways and metabolism of BCAAs could be therapeutic targets for the treatment of insulin resistance and T2DM.
